# Ethanol alters gene expression and cell organization during optic vesicle evagination

**DOI:** 10.1016/j.neuroscience.2013.07.036

**Published:** 2013-10-10

**Authors:** A. Santos-Ledo, F. Cavodeassi, H. Carreño, J. Aijón, R. Arévalo

**Affiliations:** a Departamento de Biología Celular y Patología, IBSAL-Instituto de Neurociencias de Castilla y León, Universidad de Salamanca, Spain; b Department of Cell and Developmental Biology, UCL, UK

**Keywords:** hpf, hours post-fertilization, ISH, *in situ* hybridization, MET, mesenchymal–epithelial transition, MHB, midbrain–hindbrain boundary, *oep*, *one-eye pinhead*, qRT-PCR, quantitative real-time polymerase chain reaction, SEM, standard error of mean, ss, somites stage, TUNEL, terminal deoxynucleotidyl transferase dUTP nick end labeling, ZO-1, zonula-occludens-1, eye specification, morphogenesis, cell polarity, cyclopic mutants

## Abstract

Ethanol has been described as a teratogen in vertebrate development. During early stages of brain formation, ethanol affects the evagination of the optic vesicles, resulting in synophthalmia or cyclopia, phenotypes where the optic vesicles partially or totally fuse. The mechanisms by which ethanol affects the morphogenesis of the optic vesicles are however largely unknown. In this study we make use of *in situ* hybridization, electron microscopy and immunohistochemistry to show that ethanol has profound effects on cell organization and gene expression during the evagination of the optic vesicles. Exposure to ethanol during early eye development alters the expression patterns of some genes known to be important for eye morphogenesis, such as *rx3/1* and *six3a*. Furthermore, exposure to ethanol interferes with the acquisition of neuroepithelial features by the eye field cells, which is clear at ultrastructual level. Indeed, ethanol disrupts the acquisition of fusiform cellular shapes within the eye field. In addition, tight junctions do not form and retinal progenitors do not properly polarize, as suggested by the mis-localization and down-regulation of *zo1*. We also show that the ethanol-induced cyclopic phenotype is significantly different to that observed in cyclopic mutants, suggesting a complex effect of ethanol on a variety of targets.

Our results show that ethanol not only disrupts the expression pattern of genes involved in retinal morphogenesis, such as *rx3* and *rx1*, but also disrupts the changes in cell polarity that normally occur during eye field splitting. Thus, ethylic teratology seems to be related not only to modifications in gene expression and cell death but also to alterations in cell morphology.

## Highlights

• Ethanol alters eye morphogenesis at early stages of embryogenesis.
• The expression patterns of some genes important for eye morphogenesis are perturbed.
• Ethanol is related to alterations in cell morphology.
• Ethanol interferes with the optic vesicles evagination.

## Introduction

Optic vesicle evagination is the process by which a unique morphogenetic domain, the eye field, gives rise to two symmetric domains. These domains then evaginate to generate the optic vesicles (Rembold et al., 2006). In zebrafish optic vesicle evagination begins at 2–3 somite stage (ss), when the medially located eye field acquires a bi-lobed shape. At this stage of development, the eye field is delimited anteriorly by the telencephalon and posteriorly by the hypothalamus anlage (England et al., 2006), organization that is partially inverted at later stages. Just 4 h later (at 10 ss), two optic primordia are distinguishable in the lateral parts of the embryo. The exact mechanism by which the eye field splits in two domains and the optic vesicles evaginate remains un-clear. Some studies suggest that retinal progenitors actively migrate toward lateral regions during eye morphogenesis and that this process, regulated by the transcription factor *rx3*, provides the driving force for optic vesicle evagination (Rembold et al., 2006). Other studies instead suggest that the cells within the eye field do not actively migrate, but follow the morphogenetic reorganizations promoting forebrain morphogenesis (England et al., 2006).

Many transcription factors are involved in the specification of the eye field, including *otx2*, *pax6*, *rx3*, *six3* and *zic2* among others (review in Bailey et al., 2004; Zaghloul and Moody, 2007). These transcription factors are coincidently expressed in the eye field, and their combined activity is sufficient to induce eye fate. Indeed, ectopic eyes are induced when a cocktail of these factors is ectopically expressed outside of the neural plate (Zuber et al., 2003). The molecular mechanisms involved in the morphogenesis of the eye field are not so well understood, but some reports suggest that the same genes that control eye field specification subsequently control its morphogenesis. For example, the absence of *rx3* leads to a failure in the splitting of the eye field and results in complete absence of the optic vesicles, a phenotype known as anophthalmia (Mathers et al., 1997; Winkler et al., 2000; Kennedy et al., 2004). Mutations on *six3* or *zic2* lead to holoprosencephaly and cyclopia (partially fused optic vesicles) in humans (Brown et al., 1998; Pasquier et al., 2000), also suggesting a role of these genes in the morphogenetic reorganization underlying optic vesicle evagination.

In addition to genetic factors, drugs like cyclopamine, forskolin or ethanol can also result in micro/anophthalmic and cyclopic phenotypes (Arenzana et al., 2006; Loucks et al., 2007; Santos-Ledo et al., 2011). The aim of this work is the analysis of the molecular and cellular mechanisms underlying ethanol-induced cyclopia. This teratogenic substance induces a constellation of problems during development such as delayed differentiation, increased apoptosis or migration failures, among others (Blader and Strähle, 1998; Loucks et al., 2007). The developing visual system is very sensitive to exposure to ethanol (Kashyap et al., 2007; Santos-Ledo et al., 2011) but there is no agreement about how this drug induces cyclopic phenotypes. The most prevalent model states that ethanol disrupts the collective migration of prechordal plate progenitors to the anterior part of the embryo, leading to cyclopia (Blader and Strähle, 1998). On the other hand, some studies have shown a rescue of the cyclopic phenotype by exposing zebrafish embryos to substances such as Shh (Loucks and Ahlgren, 2009) or retinoic acid (Marrs et al., 2010). However, the behavior of eye field cells after exposure to ethanol has not been analyzed.

In this study, we have analyzed the expression pattern of genes known to be involved in eye field specification and morphogenesis (*otx2*, *zic2*, *pax6*, *six3*, *rx3* and *rx1*) after exposure to ethanol. We have also analyzed the cytoarchitecture of the eye field during the early stages of eye morphogenesis and the distribution and expression levels of zonula-occludens-1 (ZO-1), a protein involved in tight junction formation and apico-basal cell polarization. Our results suggest that ethanol not only alters the expression patterns of some of the genes important for eye formation, but also prevents the cellular rearrangements that normally occur during optic vesicle evagination. Since up to now the effect of ethanol on cell morphology had only been studied in cell culture (Guasch et al., 2007; Martínez et al., 2007), our results expand our understanding of ethylic teratology *in vivo*, and suggest that ethanol-induced phenotypes result from a combination of molecular defects on both gene function and cell morphology.

## Experimental procedures

### Specimens and ethanol treatment

AB zebrafish strain embryos were used in all the experiments. They were obtained by natural pair-wise mating and staged and reared according to standard procedures (Westerfield, 1995). A previous work in our lab showed that this strain is sensitive to ethanol and a concentration of 1.5% is enough to consistently produce cyclopia (Arenzana et al., 2006).

All of the following procedures were carried out in un-treated control embryos and embryos exposed to different concentrations of ethanol: 0.5%, 1%, 1.5%, 2% and 2.4%. Embryos were exposed from dome/30% epiboly [4.3 hours post-fertilization (hpf)] to tailbud stage (10 hpf), then they were washed out of the ethanol and developed to the desired stage. Embryos were collected at 3 ss (11 hpf, prior to evagination), 6 ss (12 hpf, mid-evagination), 10 ss (14 hpf, two optic primordial are distinguishable) and 18 ss (18 hpf, optic cup formation). This protocol is similar to others previously described where cyclopic phenotypes were analyzed and a dose-dependent response of ethanol was assessed (Arenzana et al., 2006; Loucks et al., 2007).

The mutant lines *cyclops*, *one-eyed pinhead* and *trilobite* were obtained from the zebrafish Stock Centre at UCL and *silberblick* mutants were a generous gift from Dr. Masazumi Tada.

All procedures and experimental protocols were in accordance with the guidelines of the European Communities Directive (86/609/EEC and 2003/65/EC) and the current Spanish legislation for the use and care of animals in research (RD 1201/2005, BOE 252/34367-91, 2005) and conformed to NIH guidelines.

### Semi-thin sections and electron microscopy

Semi-thin sections were obtained as previously reported (Santos-Ledo et al., 2011). Briefly, embryos were fixed by immersion in 2% paraformaldehyde and 2% glutaraldehyde in 0.1 M cacodylate buffer at pH 7.4 (PB) for 24 h at 4 °C, and postfixed in osmium tetroxide containing 1% potassium ferricyanide for 1 h. Specimens were dehydrated using a graded series of cold ethanol and embedded with EMbed-812 (Electron Microscopy Science, Fort Washington, PA, EE.UU). Coronal serial sections of 1-μm-thickness were cut on an ultramicrotome Reichert-Jung Ultracut E (Nussloch, Germany). Sections were stained with 1% Toluidine Blue solution.

The same blocks were used to obtain ultra-thin sections for electron microscopy. 70-nm-thickness sections were cut in the ultramicrotome. Sections were counter-stained with 2% of uranil acetate during 15 min in darkness at room temperature and with lead citrate during 10 min at room temperature and without CO_2_. Sections were washed with distilled water and dried before observation in the electron microscope.

### *In situ* hybridization (ISH) and immunohistochemistry

ISH was performed by using published protocols (Thisse and Thisse, 2008) in embryos at 3, 6, 10 and 18 ss. Digoxigenin-labeled RNA probes were synthesized using a DIG labeling kit (Roche, Barcelona, Spain) and probes were detected with anti-DIG-AP antibody (1:5000, Roche, Barcelona, Spain) and NBT/BCIP substrate.

Whole-mount immunohistochemistry was performed as previously described (Wilson et al., 1990) using an anti-ZO-1 antibody (1:250, Invitrogen, Carlsbad, CA, U.S.A.) that has been previously tested in zebrafish (Zhang et al., 2010). Nuclei were counterstained using sytox Orange (1:10,000, Invitrogen, Carlsbad, CA, U.S.A.) or DAPI (1:10,000, Sigma, St. Louis, MO, EE.UU).

### Cell death assay

Terminal deoxynucleotidyl transferase dUTP nick end labeling (TUNEL) labeling to detect apoptosis in whole-mount embryos was performed using the ApopTag Kit (Chemicon International, Temecula, CA, USA) and a Cy3-conjugated IgG Fraction mouse anti-DIG antibody (1/500, Jackson ImmunoResearch, Oaks Drive Newmarket, Suffolk, UK). The embryos were sectioned on a cryostat and viewed using a photomicroscope. The total number of positive cells in three non-consecutive sections of six different embryos at 75% epiboly (8 hpf), three, six and 18 somites were counted. TUNEL results are expressed as mean ± SEM (standard error of mean). The mean of the number of transcripts from each experimental group was compared with the mean of the number of transcripts from the control group using Student’s *t*-test.

### Quantitative real-time polymerase chain reaction (qRT-PCR)

Quantification of expression levels of *zo-1* was determined in embryos from the six experimental groups (control and ethanol exposed) at 10 ss. Total RNA was extracted using Trizol® Reagent (Invitrogen, Carlsbad, CA, U.S.A.). cDNA synthesis was carried out by reverse transcription of total RNA to cDNA using the Applied Biosystems (Foster City, CA, USA Sincerely, Rosario Arévalo) KIT following the instructions of the manufacturer.

The concentration of cDNA was determined by measuring the absorbance at 260 nm with a NanoPhotometer™ (Implen, Germany). The quantification of the PCR products was performed using the SYBR-Green method as previously described (Sánchez-Simón and Rodríguez, 2008). The oligonucleotides used to amplify *zo-1* were: Zfzo1-F: ATCTTACGGCCGAGCATGAA; Zfzo1-R: GAGAATCTGGTCTCCCTCT. PCR products were amplified in an ABI Prism 7300 detection system (Applied Biosystems), with the following conditions: 10 min at 95 °C followed by 35 cycles of 10 s at 95 °C and 1 min at 60 °C. Three different samples have been used in the qPCR experiments and each experiment has been repeated three times. EF1 was used as internal control.

qPCR results are expressed as mean ± SEM. The mean of the number of transcripts from each experimental group was compared with the mean of the number of transcripts from the control group using an analysis of variances (one-way analysis of variance (ANOVA)) together with Dunnett’s post-test.

### Image analysis

Semi-thin sections were examined under a compound microscope Leica Aristoplan with brightfield condensers. The background was controlled and the photomultiplier voltage (800 V) selected for maximum sensitivity in the linear range. Digital images were obtained with an Olympus OP-70 digital camera (Olympus Corporation, Tokyo, Japan) coupled to an Olympus Provis AX70 photomicroscope. Sharpness, contrast, and brightness were adjusted to reflect the appearance seen through the microscope.

Ultra-thin sections were visualized in an electron microscope ZEISS-EM 900 with a TRS camera (Slow Scan CCD) and the images were taken with the ImageSP Viewer software.

Whole-mount ISH embryos were dehydrated in glycerol and images were obtained using a microscope Leica M165FC with a Leica DFC 500 camera using the Leica Application Suite V3 software.

ZO-1 immunohistochemistry images were obtained with a laser scanning spectral confocal microscope (Leica TCS SP2) using excitation of fluorochromes with a laser wavelength of 488 nm and a filter-free prism spectrophotometer. The original images were processed digitally with Adobe® Photoshop® CS5 software (Adobe Systems, San Jose, CA, U.S.A).

## Results

### The expression of genes required for eye specification and morphogenesis is altered by exposure to ethanol

We have analyzed the expression pattern of some of the transcription factors involved in the early stages of visual system development, candidates to be altered by exposure to ethanol. From the battery of genes analyzed, three were altered within the eye field (*six3a* and *rx3* and *rx1*), two of them were unaffected (*otx2* and *zic2a*) and one of them was altered but not in the eye field (*pax6a*).

**Fig. 1:**
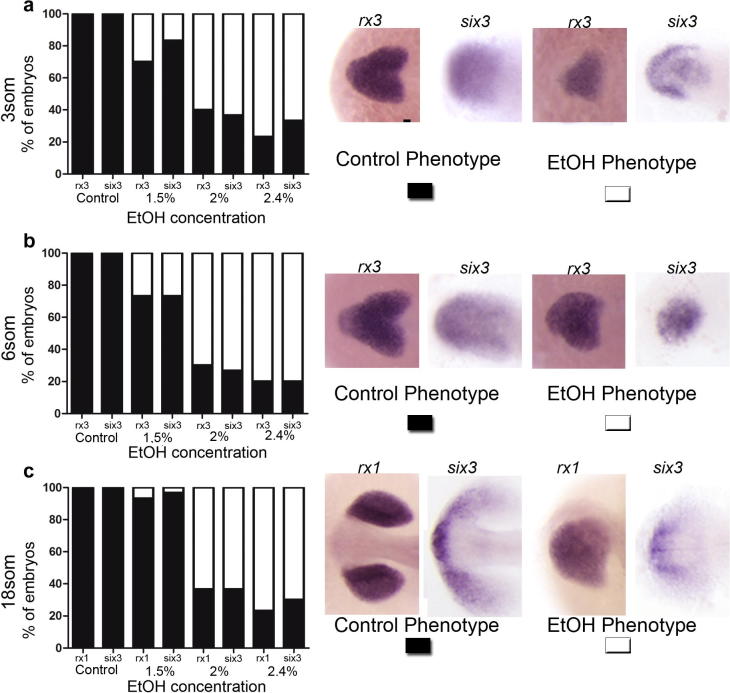
Expression patterns of *rx3, rx1* and *six3* in embryos at 3 ss (a), 6 ss (b) and 18 ss (c). These genes show an altered expression (ethanol phenotype). Quantifications on the number of embryos that show control and ethanol phenotypes in each stage and treatment are shown in the graphs at the left. Scale bar = 100 μm.

*rx3* expression is specific to the eye field and starts at mid-gastrula stage. At 3 ss the eye field shows a heart-shaped appearance, with a caudal indentation reflecting the progression of the splitting of the eye field (Fig. 1a). Exposure to ethanol disrupts this pattern so that the caudal indentation is lost, reflecting a perturbation of the earliest stages of eye morphogenesis. The number of embryos that display this phenotype is dose-dependent (20% of the embryos exposed to 1.5% of ethanol, 60% of those exposed to 2% and 80% of those exposed to 2.4% of ethanol). At 6 ss the expression pattern is similar but the caudal indentation has progressed further to the anterior part of the embryo (Fig. 1b). Again, this indentation is not observed after exposure to ethanol and the proportions of the embryos that display the ethanol phenotype are similar to those found at 3 ss. From 10 ss onward, *rx3* expression is down-regulated and it is substituted by *rx2* and *rx1*. Optic vesicle evagination has finished in control animals, and two optic primordia can be clearly distinguished by 18 ss (Fig. 1c). In the embryos exposed to ethanol the optic vesicles do not evaginate appropriately, remaining fused at the midline.

The expression of *six3a* is also perturbed after exposure to ethanol. Similarly to the effect on *rx3* and *rx1*, the number of embryos that show an ethanol phenotype increases in a dose-dependent manner. At 3 ss, *six3a* is expressed in the prosencephalon, including the eye field (Fig. 1a) but after exposure to ethanol there is a reduction in the levels of expression of this gene. *six3a* expression is similarly reduced in ethanol-exposed embryos as compared to wild type at 6 ss (Fig. 1b). At 18 ss, the expression of *six3a* is normally detected in the optic stalk, the optic vesicles and the hypothalamus anlage (Fig. 1c). In embryos exposed to ethanol the staining within the optic vesicles is reduced, whereas the staining in the other domains is maintained. The proportion of embryos with ethanol-induced phenotype is similar to that observed at early stages and also to the effect on *rx1* after exposure to ethanol (Fig. 1a–c).

**Fig. 2:**
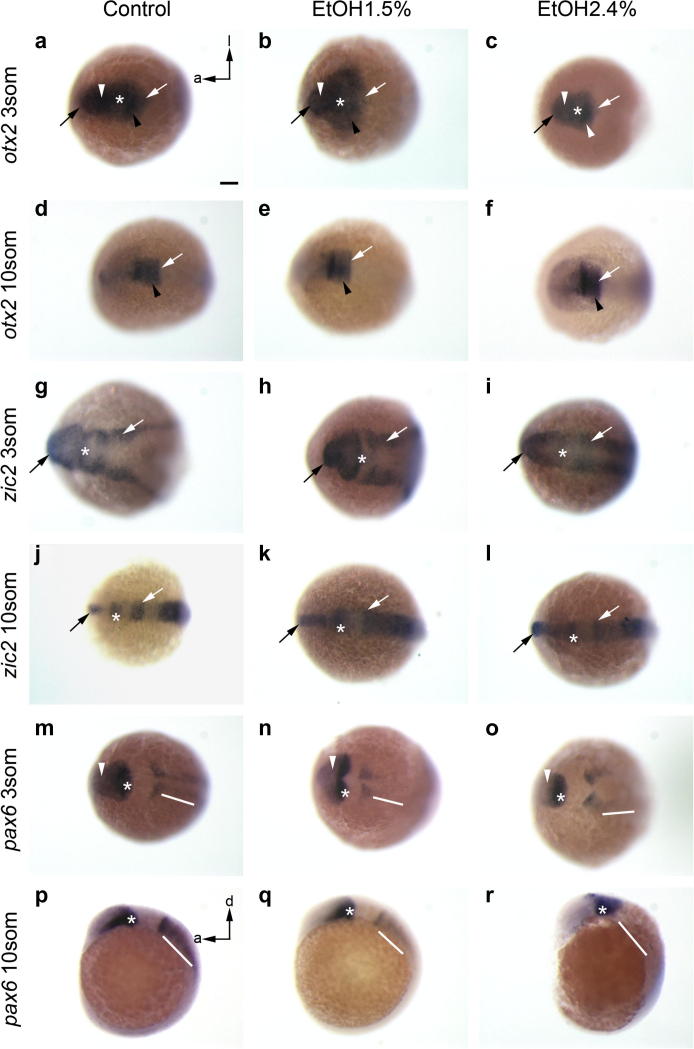
Expression pattern of *otx2* (a–f), *zic2* (g–l) and *pax6* (m–r). None of these genes is altered in the anterior part of the embryo. *pax6* expression is down-regulated in the region of the hindbrain and anterior spinal cord after exposure to ethanol (p–r). a: anterior; d: dorsal; l: lateral. Asterisk: diencephalon; black arrow: telencephalon; black arrow-head: midbrain; white arrow: midbrain–hindbrain boundary; white arrow-head: eye field; white line: hindbrain and anterior spinal cord. Scale bar = 100 μm.

Thus, ethanol treatments extensively disrupt the expression the expression of *rx3*, *rx1* and *six3a*. It is unlikely that this effect is due to general patterning defects during neural plate regionalization, since the pattern of other transcription factors important for eye formation such as *otx2*, *pax6a* or *zic2a*, is not affected in these conditions (Fig. 2)*.*

*otx2* function is essential to specify the eye field (Kenyon et al., 2001) and its expression at 3 ss is normal in all our experimental groups. *otx2* is expressed in the most anterior part of the embryo, including the eye field, the telencephalon, the diencephalon and the midbrain (Fig. 2a). This pattern was essentially unchanged after treatment with ethanol, although at lower concentrations of ethanol (1.5%) there is a medio-lateral expansion of the domain (Fig. 2b), likely due to ethanol-induced convergence-extension problems during gastrulation. At higher concentrations of ethanol (2.4%) we observed a slight reduction in the levels of *otx2* expression (Fig. 2c). At 10 ss, the expression of *otx2* in wild type embryos is down-regulated in the eye field (Andreazzoli et al., 1999) and expression becomes restricted to the midbrain, the midbrain–hindbrain boundary (MHB) and the most anterior part of the hindbrain (Fig. 2d). There are no changes in this pattern of expression when embryos are exposed to ethanol (Fig. 2e, f).

The transcription factor z*ic2a* has been shown to maintain the multipotent state of neural cells (Brown and Brown, 2009) and is prominently expressed in the forebrain. At 3 ss *zic2a* is detected in the telencephalon, diencephalon, MHB and the lateral limits of the midbrain and hindbrain (Fig. 2g). At 10 ss, *zic2a* is expressed in the telencephalon, diencephalon, MHB and posterior neural plate, but there is no expression within the midbrain and the hindbrain (Fig. 2j). We detect a slight delay in the refinement of *zic2a* expression pattern after exposure to ethanol but differences are recovered by 10 ss (Fig. 2h–l). This delay is consistent with the general delay produced by ethanol throughout development (Blader and Strähle, 1998).

*pax6a* is also essential during the development of the visual system (Zaghloul and Moody, 2007). At 3 ss, this gene is expressed in the diencephalon and the eye field, the MHB and two longitudinal stripes in the hindbrain (Fig. 2m). At this stage no changes are observed after exposure to ethanol (Fig. 2n, o) except from a curvature in the most posterior domain caused by convergent extension defects. At 10 ss, *pax6a* is expressed in the dorsal diencephalon and along the ventral diencephalon, up to the most anterior part of the forebrain (Fig. 2p). In ethanol treated embryos the ventral diencephalic expression is lost and the expression in the hindbrain is reduced in a dose-dependent manner (Fig. 2q, r).

**Fig. 3:**
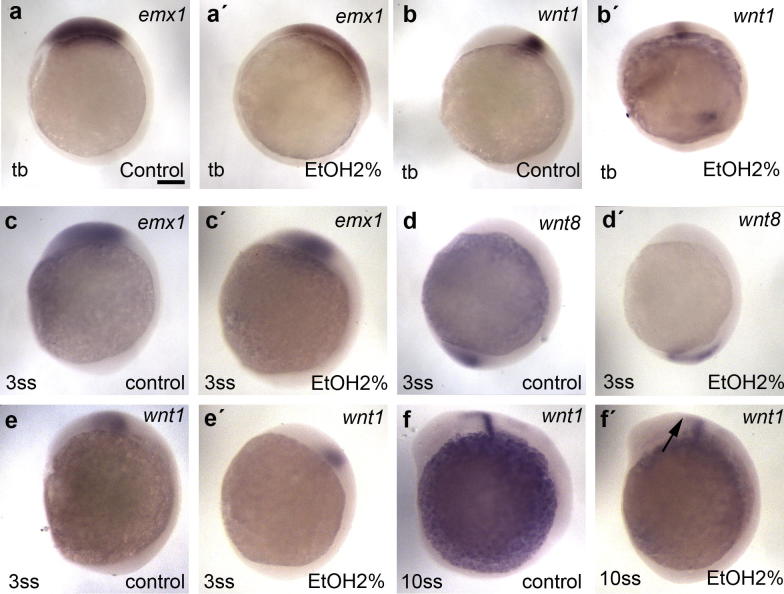
Expression pattern of *emx1*, *wnt1* and *wnt8* at different stages in control animals and in embryos exposure to ethanol. Scale bar = 100 μm.

To further assess whether the changes observed in the eye field could be extended to other regions, we analyzed the telencephalic marker *emx1* and *wnt1*, which is involved in hindbrain specification at gastrula stage. *emx1* shows an expanded expression (Fig. 3a, a′) consistent with the previously reported defects in convergent and extension movements (Blader and Strähle, 1998). *wnt1* shows a fainter expression, slightly mislocated (Fig. 3b, b′). During early somoitogenesis stages the reduction of *emx1* (Fig. 3c, c′) and the expansion of *wnt8* (Fig. 3d, d′) expression suggest a posteriorization of the embryo, likely as a consequence of the gastrulation defects and the defective migration of the prechordal plate progenitors that remain in a more posterior location (Blader and Strähle, 1998). *wnt1* is also slightly reduced in its expression at three somites (Fig. 3e, e′) and aberrantly expanded throughout the midbrain at 10 somites (Fig. 3f, f′).

### The cytoarchitecture and cell polarity of the optic vesicles is highly perturbed by ethanol treatment

Exposure to ethanol alters the expression pattern of some of the genes involved in eye field specification and morphogenesis. Nevertheless, it is not clear if these alterations are the cause of the morphogenesis defects. Since it has been described that retinal progenitors require *rx3* for elongation during eye morphogenesis (Medina-Martínez et al., 2009) we decided to analyze the cytoarchitecture of the eye field. We have restricted our morphological analysis to embryos exposed to 2% of ethanol because it has been shown that this concentration produces a consistent proportion of cyclopic embryos (Arenzana et al., 2006) and induces fewer un-related problems than 2.4%.

**Fig. 4:**
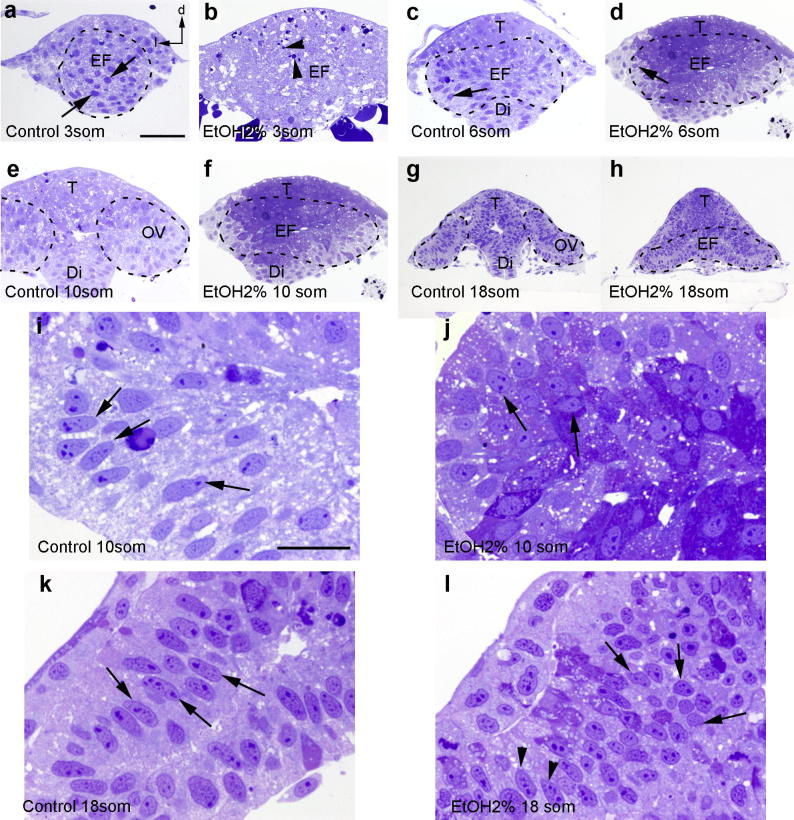
Cytoarchitecture of the eye field. At 3 ss retinal progenitors present big nuclei and circular shape (arrows in a), but not in the ethanol-treated embryos (b) which present several pyknotic nuclei (arrowhead in b). At 6 ss, retinal progenitors have fusiform morphologies only in control animals (arrows in c and d). From 10 ss onward, two optic vesicles are distinguishable only in un-treated animals (e–h). Retinal progenitors show an elongated morphology in control animals (arrows in i, k) compared to the circular shape in embryos exposed to ethanol (arrows in j, l), that only have them in the ventral part of the retina (arrowheads in l). d: dorsal; Di: diencephalon; EF: eye field; l: lateral; OV. Optic vesicle; T: telencephalon. Scale bar: a–h = 50 μm; i–l = 20 μm.

Eye field cells in 3-ss embryos have a round shape, big nuclei and small inter-cellular spaces (Fig. 4a). The eye field is clearly distinguishable in wild-type embryos, yet it is not easily found after exposure to ethanol and many pyknotic nuclei and bigger inter-cellular spaces are observed (Fig. 4b). At 6 ss the eye field is partially split in control animals and retinal progenitors have elongated and acquired fusiform morphologies (Fig. 4c). In the embryos exposed to ethanol the eye field can be recognized but these changes in cell morphology are not so evident (Fig. 4d).

By 10 ss the two optic vesicles are totally separated in control embryos, and eye cells show a fusiform appearance (Fig. 4e, i). Between the optic vesicles, the telencephalon and the diencephalon can be distinguished. In the embryos exposed to 2% of ethanol the optic vesicles remain fused and some of the cells are elongated but in a disorganized way (Fig. 4f, j). Most of the progenitors are smaller and rounder than in control animals (Fig. 4i, j). At 18 ss, the optic cup is being formed, the eye field progenitors are not directly joined to the prosencephalon but through the optic stalk and retinal progenitors are elongated (Fig. 4g, k). In embryos exposed to ethanol the eye vesicles remain fused in the midline (Fig. 4h). Although some retinal progenitors have acquired elongated morphologies, many others are still round and small, especially in the dorsal part of the eye field (Fig. 4h, l).

Thus, the elongated and fusiform shape of wild-type retinal progenitors (Fig. 4i) is lost after exposure to ethanol and many eye cells maintain a round morphology; those that acquire an elongated shape do not orient in a specific pattern (Fig. 4j). These differences are maintained at least until 18 ss (Fig. 4k, l), although some recovering occurred in the ventral part of the eye field where some cells eventually elongate (Fig. 4l).

**Fig. 5:**
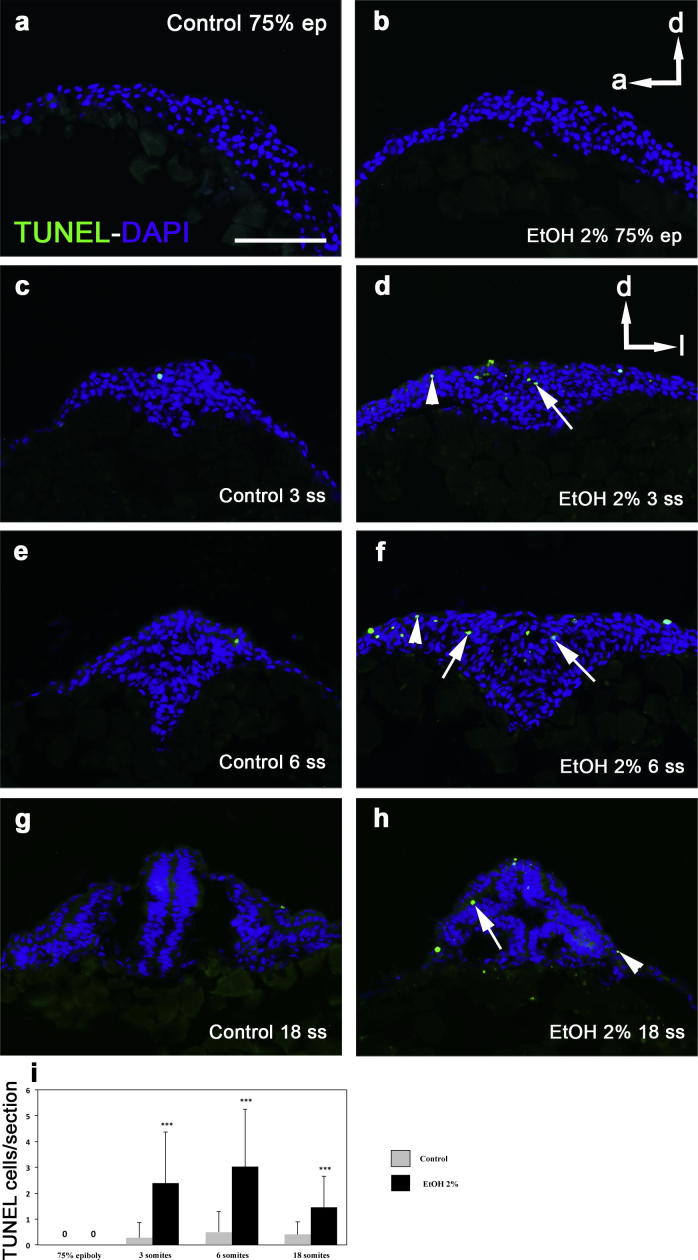
Cell death assay by TUNEL at 75% epiboly (a, b), three (c, d), six (e, f) and 18 (g, h) somites. The number of positive cells (i) is higher in embryos exposed to ethanol at three, six and 18 somites both within the eye field (arrows) and in other regions of the anterior part (arrowheads). ^∗∗∗^*P* < 0.0001.

We have previously reported that exposure to ethanol induces an increase in cell death at later stages (Arenzana et al., 2006). To check if the pyknotic nuclei correlate with cell death we performed cell death assay by TUNEL and quantified the total number of positive cells per sections. No cell death was observed at 75% epiboly, the stage at which the prechordal plate progenitors reach the anterior part of the embryo (Fig. 5a, b, i). Cell death is present in control animals at 3 ss (Fig. 5c), 6 ss (Fig. 5e) and 18 ss (Fig. 5g) at very low levels (Fig. 5i). The number of TUNEL-positive cells is significantly higher in embryos exposed to ethanol (Fig. 5d, f, h, i) and they are not only within the eye field (arrows in Fig. 5d, f, h) but also in other regions of the anterior part of the embryo (arrowheads in Fig. 5d, f, h). The effect is more obvious and similar at 3 and 6 ss, while there is a partial amelioration of the cell death at 18 somites.

**Fig. 6:**
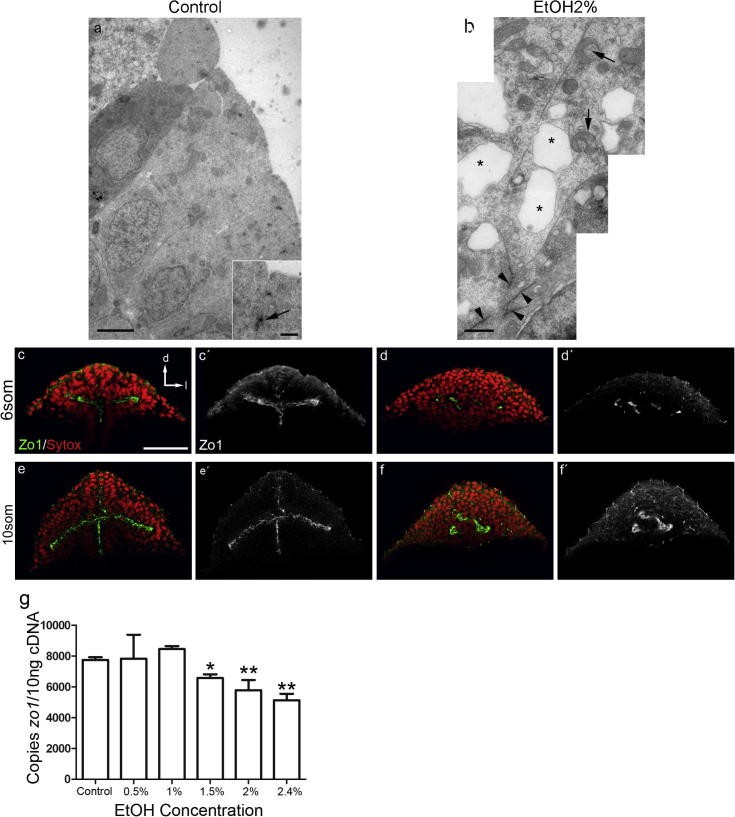
(a, b) Electron microscopy images from retinal precursor of 10-ss embryos. Retinal progenitors within the eye field display epithelial characteristics (a) with many well-organized junctions (arrow in inset in a). Exposed embryos present mitochondrias with aberrant morphologies (arrows in b), low density of ribosomes, big vacuoles (asterisks in b) and disorganized cell junctions (arrowheads in b). (c–f′) ZO-1 is located in the apical part of the cells at 6 (c, c′) and 10 ss (e, e′) and cells are oriented toward a mid axis. After exposure to ethanol, ZO-1 staining is dispersed and there is no organization of a mid axis (d, d′, f, f′). (g) qRT-PCR of *zo1* expression at 10 ss. Exposure to ethanol down-regulates *zo1* expression in a dose-dependent manner. d: dorsal, l: lateral. Scale bar: a = 2500 nm; inset in a, b = 500 nm; c–f′ = 50 μm. ^∗^*P* < 0.05; ^∗∗^0.05 > *P* > 0.001.

At electron microscopy level, retinal progenitors show the typical aspect of epithelial cells: elongated, with the nuclei located basally, prominent intercellular junctions and high density of ribosomes (Fig. 6a). The retinal progenitors of embryos exposed to ethanol present instead several vacuoles of different sizes, mitochondria with aberrant morphologies, less density of ribosomes and, although they also present intercellular junctions, they are mislocated (Fig. 6b).

Since the cell morphology and intercellular junctions of retinal progenitors were perturbed we wondered whether these cells were polarizing appropriately. ZO-1 is a protein involved in the establishment of the apico-basal polarity and a main component of different types of cell junctions. At 6 and 10 ss, the retinal progenitors of control animals present their apical domain toward the center of the forming optic vesicles, outlining the lumen of this structure (Fig. 6c, c′, e, e′). Eye cells in the embryos exposed to ethanol are also polarized, but the staining for ZO-1 is reduced and highly disorganized (Fig. 6d, d′, f, f′). These defects are not specific to the eye field, other regions such as the telencephalon surrounding the eye field also show problems in cell polarity. While in control animals, polarized cells can be only observed in the central part of the telencephalon (Fig. 6e, e′), in embryos exposed to ethanol, this pattern cannot be recognized and scattered polarized cells are observed all over the telencephalon (Fig. 6f, f′).

In the immunostained embryos, ethanol seemed to reduce the levels of ZO-1. To check whether this was actually the case, we quantified the amount of ZO-1 transcripts by qRT-PCR in control versus ethanol treated embryos. Exposure to ethanol significantly reduces the expression of *zo1* when a 1.5% concentration of ethanol is used, a concentration that has been shown to consistently produce cyclopic embryos in this strain (Arenzana et al., 2006). Moreover, the ethanol-induced down-regulation of *zo1* is dose dependent (Fig. 4g).

### Cyclopic mutants show a qualitatively different phenotype to that induced by ethanol

Several mutants in components of the Nodal and Wnt pathways have been identified over the years with cyclopic phenotypes. We compared the cellular organization of some of these cyclopic conditions with that of ethanol-treated embryos, by analyzing the distribution pattern of ZO-1 in them.

**Fig. 7:**
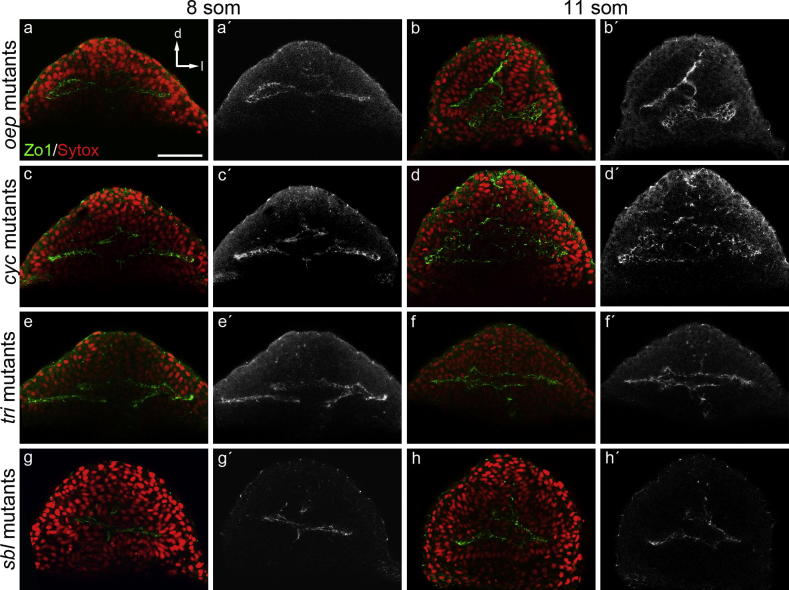
Distribution pattern of ZO-1 in zebrafish mutants *oep* (a–b′), *cyc* (c–d′), *tri* (e–f′) and *slb* (g–h′). All of them show an aberrant accumulation of ZO-1, however the phenotype is qualitatively different from that observed in ethanol treated embryos. d: dorsal, l: lateral. Scale bar: a–h′ = 50 μm.

*One-eye pinhead* (*oep*) encodes a co-receptor of Nodal signals, and shows a completely cyclopic phenotype (Hammerschmidt et al., 1996). *oep* mutants show an apparently normal eye field at 8 ss stage with a clear expression of ZO-1 (Fig. 7a, a′). At later stages, the eye field did not evaginate properly and only one eye is evident, although retinal progenitor cells’ organization in this single eye is completely normal (Fig. 7b, b′).

*cyclops* (*cyc*, encoding a Nodal ligand, Macdonald et al., 1995), *trilobite* (*tri*, encoding the non-canonical Wnt pathway component *van-gogh*, Hammerschmidt et al., 1996) and *silberblick* (*slb*, encoding the non-canonical Wnt ligand *wnt11*, Heisenberg et al., 1996) all present a similar pattern of ZO-1 expression. At 8 ss retinal progenitors are polarized toward the lumen but a delay in the evagination is already seen (Fig. 7c, c′, e, e′, g, g′). At 11 ss retinal progenitors are oriented toward the central part of the embryo but between the dorsal and the ventral parts of the unique eye field there is a clump of misoriented cells (Fig. 7d, d′, f, f′, h, h′).

Thus, whereas all these mutant conditions show cell polarization defects during optic vesicle evagination, they present a more or less organized lumen and most of the cells within the eye field polarize. After exposure to ethanol we observe a qualitatively different phenotype, where the lumen never forms and cell polarity markers are significantly reduced. This suggests that the phenotypic consequences of exposure to ethanol are not a simple consequence of an effect of ethanol on the activity of the Nodal or Wnt pathways.

## Discussion

### Ethanol and gene expression

In zebrafish the evagination of the optic vesicles occurs between 3 and 10 ss. This process is perturbed by exposing embryos to ethanol during gastrulation, just prior to the onset of eye morphogenesis. The failure in the migration of the prechordal progenitors and the subsequent posteriorization of the embryos has been suggested as the main cause of the ethanol-induced cyclopia (Blader and Strähle, 1998). The alterations in *emx1* and *wnts* expression patterns that we observed in our analysis are consistent with this interpretation. These alterations are maintained during the splitting of the eye field, which implicates abnormal formation of brain regions such as the telencephalon and diencephalon, regions that are directly involved in the splitting of the eye field (England et al., 2006).

In addition, we have found profound alterations in the expression patterns of *six3a*, *rx3* and *rx1*, some of the most important genes for early stages of eye development. These alterations are consistent with previous results in another zebrafish strain and in embryos exposed to forskolin, a substance that also induces a cyclopic phenotype (Loucks et al., 2007). The total lack of *rx3* in *chk* zebrafish mutants leads to anophthalmic phenotypes (Kennedy et al., 2004) but *rx3* may have multiple roles during the splitting of the eye field: it controls cell proliferation and the size of the optic vesicles (Loosli et al., 2001), modulates the convergence and lateral migration of retinal progenitors (Rembold et al., 2006), and controls retinal cell morphology (Medina-Martínez et al., 2009). During optic vesicle evagination, *rx3* expressing cells are displaced laterally and *rx3* expression is substituted by *rx1*. In ethanol-exposed embryos the expression of *rx1* is maintained between both optic vesicles. *rx1* contributes to the differentiation of photoreceptors at later stages of eye development (Chuang and Raymond, 2001), and indeed the region where the optic vesicles fuse in ethanol-induced cyclopic animals gives rise to many photoreceptors (Santos-Ledo et al., 2011), a phenotype also found in *cyc* mutants (Fulwiler et al., 1997). *rx1* may thus contribute to the excessive differentiation of photoreceptors in the region where the optic vesicles fuse (Santos-Ledo et al., 2011).

*six3a* expression is also reduced after exposure to ethanol. Ethanol effect is restricted to the optic vesicles, since *six3a* expression is normal in the optic stalk and in the most anterior part of the hypothalamus. Mutations in *six3a* have been frequently related to holoprosencephaly in humans (Wallis et al., 1999) and zebrafish (Domene et al., 2008), where it has been shown to alter *shh* signaling (Sanek et al., 2009). In medaka, the injection of Geminin, a protein that binds and blocks Six3, or the injection of suboptimal amounts of *six3a* morpholino can also induce cyclopic phenotypes (Carl et al., 2002; del Bene et al., 2004). Since*six3* is an important modulator of proliferation (del Bene et al., 2004), the reduction in its expression in cyclopic (Santos-Ledo et al., 2011) and microphtalmic (Kashyap et al., 2007) models may be linked to the reduction in the size of the optic vesicles in these models.

Not all the genes required for eye field specification are affected by ethanol treatments. Indeed, the expression of *otx2*, *zic2* and *pax6* is not perturbed by exposure to ethanol, suggesting that the effect of ethanol exposure on the expression of *rx* genes and *six3a* is not an indirect consequence of perturbations in anterior neural plate patterning, but rather a direct effect on the expression of those genes. The number of embryos that show altered expression of *rx3* and *six3a* depends on the concentration of ethanol used. We have shown that incubations in 2.4% of ethanol induce a gene expression phenotype in 80% of the cases, a higher percentage than the embryos actually showing cyclopia (46.5%, Arenzana et al., 2006). This observation suggests that altered gene expression, despite probably being a direct consequence of ethanol treatment, may not be the main cause of the cyclopic phenotype, and that this may lay in other mechanisms that are discussed in the next section.

### Ethanol, cell shape and cell death

We have analyzed the cytoarchitecture of the eye field during its transformation into optic vesicles, paying special attention to cell shape, cell junctions and cell polarity. In control animals there is a big change in cell shape between 3 and 6 ss. In this period of time, cells acquire fusiform morphologies and orient their apical domain to the central part of the eye field. These changes were described in the zebrafish by Schmitt and Dowling in 1994, and a similar transformation occurs in mice (Svoboda and O’Shea, 1987). After exposure to ethanol this transformation in eye cell shape is altered; many of the cells retain circular shapes and do not polarize properly.

Exposure to ethanol induces a delay in the morphogenetic changes that occur during optic vesicle evagination. Although cells partially elongate and establish some cell junctions between them, the tissue is highly disorganized. Retinal progenitors present several vacuoles, low ribosome density and mitochondria with aberrant morphologies, characteristics that were described associated to exposure to ethanol in other cell types and contexts long time ago (Rossi and Zucoloto, 1977; Bannigan and Burke, 1982), but also in the visual system more recently (Pinazo-Duran et al., 1993). Vacuoles and mitochondria are involved in ethanol detoxification (review in Manzo-Avalos and Saavedra-Molina, 2010). Moreover, ethanol releases free radicals from mitochondria (Robin et al., 2005) and reduces their function (Weiser et al., 2011). All together these alterations could result in the lack of change in cell morphology. The lack of changes in cell morphology occurs together with high levels of cell death all over the embryo. Although a correlation between cell death and defective accumulation of Zo-1 has not been shown, an increased levels of cell death could likely contribute to the cyclopic phenotype as it has been previously shown in zebrafish embryos injected with Pard3 (Wei et al., 2004) and in chicken embryos exposed to BMP (Golden et al., 1999).

During optic vesicle evagination eye cells go from showing a mesenchymal appearance to acquire epithelial characteristics. Although we have no evidence to prove that this process is a mesenchymal–epithelial transition (MET), there may be some mechanisms in common (reviewed in Holley, 2007). During MET, changes in cell fate result in the accumulation of cell adhesion complexes in a latero-apical domain, and promotes the localization of the nucleus in the basal part of the cell and the centrosome in the apical part (Barrios et al., 2003). These changes are also observed during optic vesicle evagination, including the accumulation of ZO-1 in the latero-apical part of the cells. ZO-1 is involved in the formation of different kinds of cell junctions such as zonulas ocludens and adherens (Ciolofan et al., 2006) and in absence of it cells cannot establish proper connections between them (Umeda et al., 2006). We have described a reduction in the levels of ZO-1 protein after exposure to ethanol, and an aberrant distribution of what is left, which could prevent the correct maturation of the cell junctions and the acquisition of epithelial morphology. The high levels of cell death could contribute to the reduction and miss-expression of ZO-1.

The small GTPases is a family of molecules that also controls the acquisition of epithelial shape (Nakaya et al., 2004). These molecules are key regulators of several pathways, including the polymerization and de-polymerization of Actin and the formation of cell protrusions such as filopodia and lamellipodia (review in Ridley, 2011), a feature also shown by retinal progenitors during optic vesicle evagination (Rembold et al., 2006). Cells cultured in the presence of ethanol show an altered organization of the Actin cytoskeleton and microtubules (Romero et al., 2010) and the expression of some of these small GTPases is perturbed (Guasch et al., 2003, 2007). A similar perturbation of small GTPases after exposure to ethanol may also occur in zebrafish embryos (unpublished observations).

### Ethanol and mutations

Cyclopia is a common phenotype when gastrulation is perturbed. In fact all the mutants analyzed here were discovered in mutagenesis screens related to gastrulation defects (*cyc*: Sampath et al., 1998; *oep* and *tri*: Hammerschmidt et al., 1996; *slb*: Heisenberg et al., 1996). The mechanisms underlying cyclopia are relatively well understood in *cyc* and *slb* mutants and strikingly the mechanism proposed in each case is different. In *cyc* mutant embryos cyclopia seems to be due to a failure in the specification of the hypothalamic tissue and in *sbl* it is due to reduced anterior movements of the neural keel during the splitting of the eye field (England et al., 2006). The cyclopic phenotype in *tri* mutants has been associated to deficiencies in *shh* signaling (Marlow et al., 1998) and since *oep* acts as a cofactor in TGFβ signaling (Gritsman et al., 1999) its cyclopic phenotype may have a similar origin to that observed in *cyc*. Although we cannot discount that these mechanisms partially underlie ethanol-induced cyclopia, our results suggest a different scenario. In embryos exposed to ethanol the hypothalamus is specified and the distribution pattern of ZO-1 is very different to the one observed in those mutants, suggesting that changes of cell morphology and increased cell death underlie cyclopia in ethanol-treated embryos.

### Final remarks

In order to induce cyclopia, ethanol exposure has to occur during early gastrulation. Nevertheless, ethanol is usually not eliminated from the embryo medium until the end of gastrulation, just prior to the start of optic vesicle evagination (Bradfield et al., 2006). Thus, we cannot discard that ethanol is still present during the early stages of eye morphogenesis. Moreover, the changes in gene expression that occur during gastrulation will impact the patterning specification and may contribute to the cyclopic phenotype.

Our results show that ethanol disrupts the expression pattern of some of the genes involved in forebrain patterning and retinal morphogenesis before and during the splitting of the eye field. Ethanol also increases cell death and induces changes in cell polarity that normally occur during the evagination of the optic vesicles. Our results expand our understanding of ethylic teratology *in vivo*, and suggest that ethanol-induced phenotypes result from a combination of molecular defects on gene function, cell morphology and cell death, which are significantly different from other cyclopic mutants.

## Conflict of interest statement

There are no relationships of the listed authors with an entity that has a financial interest in the subject matter discussed in our manuscript “Ethanol alters gene expression and cell organization during optic vesicle evagination” or any financial interest or financial conflict of the academic institutions (University of Salamanca and UCL) with the subject.

## Role of authors

All authors had full access to all the data in the study and take responsibility for the integrity of the data and the accuracy of the data analysis. Study concept and design: ASL, FC, RA. Acquisition of data: ASL, FC, HC. Analysis and interpretation of data: ASL, FC, HC, JA, RA. Drafting of the manuscript: ASL, FC. Critical revision of the manuscript for important intellectual content: JA, RA. Statistical analysis: ASL, JA. Obtained funding: FC, JA, RA. Administrative, technical, and material support: JA, RA. Study supervision: JA, RA.
